# Three-Dimensional Assessment of the Temporomandibular Joint Changes Following Reversed Twin Block Therapy of Patients With Skeletal Class III Malocclusion in Conjunction With the Photobiomodulation Therapy: A Randomized Controlled Clinical Trial

**DOI:** 10.7759/cureus.25897

**Published:** 2022-06-13

**Authors:** Mohamed Abdulkarim Khwanda, Ahmad S Burhan, Mohammad Y Hajeer, Mowaffak A Ajaj, Steven Parker, Fehmieh R Nawaya, Omar Hamadah

**Affiliations:** 1 Department of Orthodontics, University of Damascus Faculty of Dentistry, Damascus, SYR; 2 Department of Oral Medicine and Radiology, Leicester School of Pharmacy, De Montfort University, Leicester, GBR; 3 Department of Pediatric Dentistry, Syrian Private University Faculty of Dentistry, Damascus, SYR; 4 Department of Oral Medicine, University of Damascus Faculty of Dentistry, Damascus, SYR

**Keywords:** growing patients, skeletal class iii malocclusion, reversed twin block, gallium–aluminum–arsenide diode laser, photobiomodulation, low-level laser therapy, cone-beam computed tomography, tmj, condylar volume

## Abstract

Background: Despite the positive effect of the photobiomodulation therapy (PBMT) application on animals, the primary role of this technique on the human condyle is still unclear. Several experimental reports have shown the efficacy of PBMT in inducing cellular changes in the temporomandibular joint (TMJ) region during functional treatment of patients with skeletal deformities. Still, the lack of information about its effects on human condyles requires further studies.

Objectives: This study aimed to evaluate the effect of PBMT on the TMJ components following Class III treatment with the reversed twin block (RTB) appliance in growing patients.

Materials and Method: Forty children (12 females, 28 males) between the age of nine and eleven years with skeletal Class III were assigned randomly to the RTB group with photobiomodulation (RTB+PBMT) or the control group (RTB). The PBMT was applied to the TMJ region using an 808-nm wavelength Ga-Al-As semiconductor laser device with 5 Joules/cm^2 ^energydensity on days 1, 3, 7, and 14 of the first month. Afterwards, the irradiation was conducted every 15 days until the end of the treatment. Cone-beam computerized tomography (CBCT) images were taken before (T1) treatment and following the end of treatment (T2) to assess TMJ and skeletal changes.

Results: Condylar volume was significantly increased in the RTB group only by a mean of 287.97 mm^3^ (p<0.001). The significantly backward and upward condylar movement was observed in the RTB and RTB+PBMT groups (superior joint space (SJS): 0.26 mm, 0.15 mm; posterior joint space (PJS): 0.42mm, 0.11mm, respectively). The RTB group showed the most remarkable changes. Significant improvement of the sagittal maxilla-mandibular relationship was greater in the RTB+PBMT group compared to the RTB group (p=0.02).

Conclusion: There were no considerable differences in the condylar position after Class III treatment between the RTB and the RTB+PBMT groups. But a difference in the condylar volume was noticed between the two group.

## Introduction

Skeletal Class III is defined as a deficiency in the sagittal relation between the upper and lower jaw, which may be accompanied by maxillary hypoplasia, mandibular prognathism, or both [1]. Skeletal Class III is less common than Class I and Class II, as its prevalence ranges from 1 to 14% among patients [2].

Class III malocclusion treatment aims to correct the imbalance between the jaws by stimulating the maxilla growth and restricting the mandible development. The applied forces may affect the inner temporomandibular joint (TMJ) structures, and an adaptive remodeling may accrue in the condyle position [3]. Therefore, it is necessary to evaluate the temporomandibular joint situation carefully. Some studies have classified class III malocclusion as a potential factor in temporomandibular disorders (TMDs) [4].

Several methods have been used to treat skeletal Class III cases in growing patients, such as the face mask [5], chin cup [6], Frankel III device [7], the removable mandibular retractor (RMR) [8], and reversed twin block (RTB) [9]. Extra-oral appliances such as the face mask have been used effectively to treat Class III patients with skeletal changes [5]. However, many patients may not cooperate with these devices due to their external appearance and being too bulky. On the other hand, more cooperation can be expected with intraoral devices, such as the RTB and the RMR, which treat class III deformities by enhancing forward maxilla movement and inhabiting mandible growth with a high receptive level by patients [9,10].

Two-dimensional (2D) imagining techniques have been used to evaluate orthodontic treatment outcomes for a long time. However, it has enormous limitations since it offers only 2D information for three-dimensional (3D) structures when three-dimensional diagnosis and volumetric information are needed. 3D images such as computerized tomography (CT) and cone-beam computerized tomography (CBCT) have been used more widely as additional methods to gain precise diagnostic information when needed. Although CT is considered an accurate diagnostic imaging technique for TMJ assessment, CBCT is preferable for its lower radiation dose and lower scan time [11].

TMJ is a complex joint that responds continuously to various functional factors and forces. The condyles, one of the basic structures of the TMJ and an important center of growth, are closely related to the jaws [12]. Therefore, an accurate understanding of these structures is of the utmost importance. However, only a few studies evaluated the 3D changes of the components of the TMJ after class III treatment using CBCT imaging [13-15].

The complete correction of Class III deformities is time-consuming. In general, many interventions have been proposed to accelerate the orthodontic movement, including surgical methods [16], physical methods [17], mechanical methods [18,19], or injection of some biological factors [20]. Traditionally, sagittal skeletal discrepancies used to be treated by functional appliances without applying any acceleration methods. Recently, many experimental studies have evaluated none invasive physical approaches during class II functional therapy, such as photobiomodulation therapy (PBMT), and verified its ability to enhance chondroblastic proliferation, affecting bone and condyle growth [21,22]. Khadra et al. stated that PBMT might enhance bone formation in rat calvarial bone defects [23]. Very recently, two RCTs have been conducted to evaluate two different physical techniques in stimulating bone growth following functional treatment [24,25]. The first RCT employed low-intensity pulsed ultrasound (LIPUS) and found it very effective in growth stimulation and reducing treatment time. The second one employed low-level laser therapy (LLLT) and found it effective in stimulating condylar growth and decreased treatment time by 45%. However, no previous studies have investigated the PBMT effects on class III functional treatment in growing patients.

Therefore this trial aimed to assess the impact of accompanying PBMT on TMJ changes following skeletal Class III treatment with RTB appliance. No clinical studies have evaluated the effect of PBMT in combination with RTB appliance on the TMJ during the treatment of growing class III patients. The hypothesis null was no significantly different changes after RTB therapy, with and without adjunctive PBMT.

## Materials and methods

Trial design

This study was designed as a single-center two-arm parallel-group randomized controlled clinical trial and was written up according to the Consolidated Standards of Reporting Trials (CONSORT) statement [26]. The study was conducted in the Department of Orthodontics at Damascus university between July 2017 and March 2020. Ethical approval was obtained from the Local Research Ethics Committee at the Faculty of Dentistry of the University of Damascus (UDDS-714-02032017/SRC-1095). This trial was registered on the German Clinical Trials Register (DRKS-ID: DRKS00027535).

Sample size calculation

The sample size was calculated using the G*power 3.1.3 program (University Kiel, Germany). The following assumptions were used: A two-sample t-test, a statistical power of 85%, and a significance level of 0.05. The effect size was calculated according to a previous study and was based on the changes in the condylar position [27]. The estimation revealed the need for 38 patients. Two patients were added to compensate for potential dropouts.

Patients' recruitment

**Figure 1:**
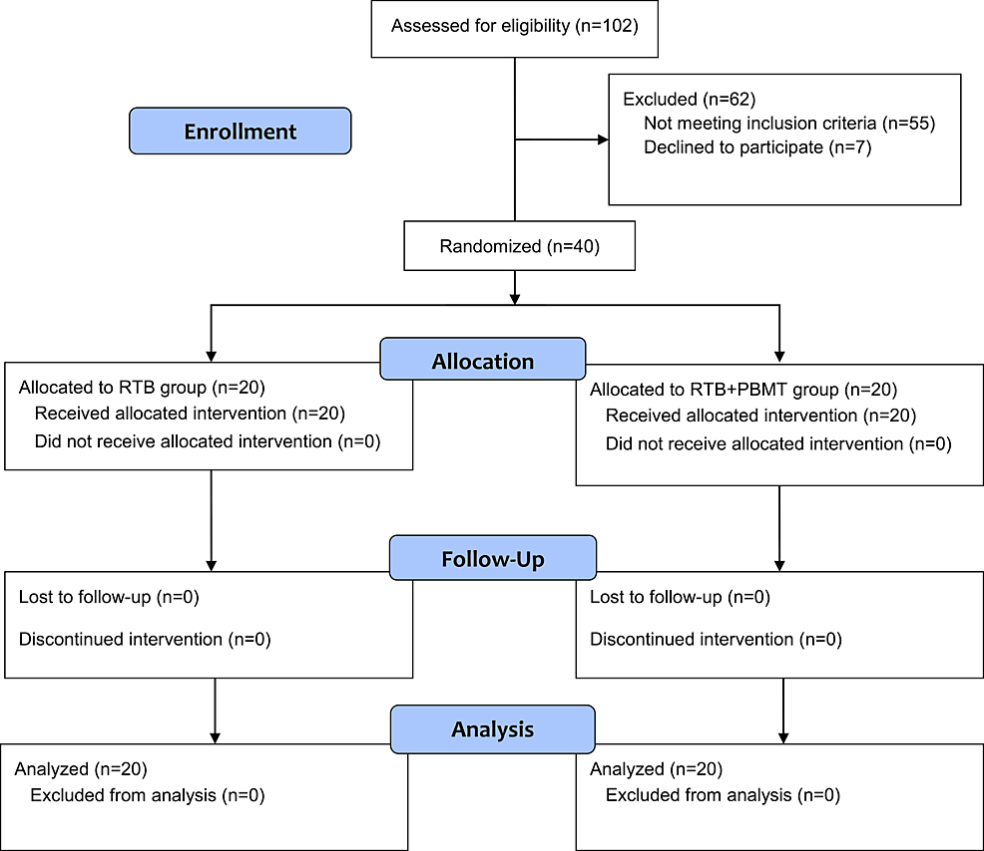
The Consolidated Standard of Reporting Trials (CONSORT) flow diagram of patients' recruitment, follow-up, and entry into data analysis.

Patients who were attending the Department of Orthodontics and Dentofacial Orthopedics at Damascus University were screened. Clinical examination was performed on 102 patients. Patients were considered eligible for the study if they met the following inclusion criteria: age between 9 and 11 years, Class III molar relationship, the presence of anterior crossbite, -3° < the sagittal skeletal discrepancy angle (SNA) angle ≤ 0°; absence of any extracted or congenitally missing teeth, no deformity in the craniofacial complex; normal or horizontal growth pattern (i.e., Sum of Bjork<400°) and no history of temporomandibular joint disorders. Exclusion criteria were: previous orthodontic treatment, vertical growth pattern, severe maxillary transverse deficiency, severe skeletal class III, severe facial asymmetry, and poor oral hygiene. Forty patients were selected to participate in this trial. The CONSORT flow diagram of patients' recruitment, follow-up, and entry into analysis is given in Figure 1. The patient's rights in this research work were protected, and informed consent forms were obtained from the patients' parents or guardians following a detailed explanation of the research project.

Randomization and blinding

Patients were assigned to the irradiation group (RTB+PBMT) or the control group (RTB) with an allocation ratio of 1:1 using a simple randomization technique. Each patient was asked to select a folded piece of paper from a box containing 40 pieces of paper; on 20 pieces, the word "RTB+PBMT group" was written, while the word "RTB" was written on the other 20 pieces. The patient was assigned to one of the two groups according to the selected paper. A member of the academic staff not involved in the research project was asked to perform the random allocation sequence generation, participants' enrollment, and assignment to the intervention. Blinding of patients or the principal researcher was impossible. Therefore, blinding was only applied in the outcome assessment.

Interventions

Reversed Twin Block (RTB) Group

**Figure 2:**
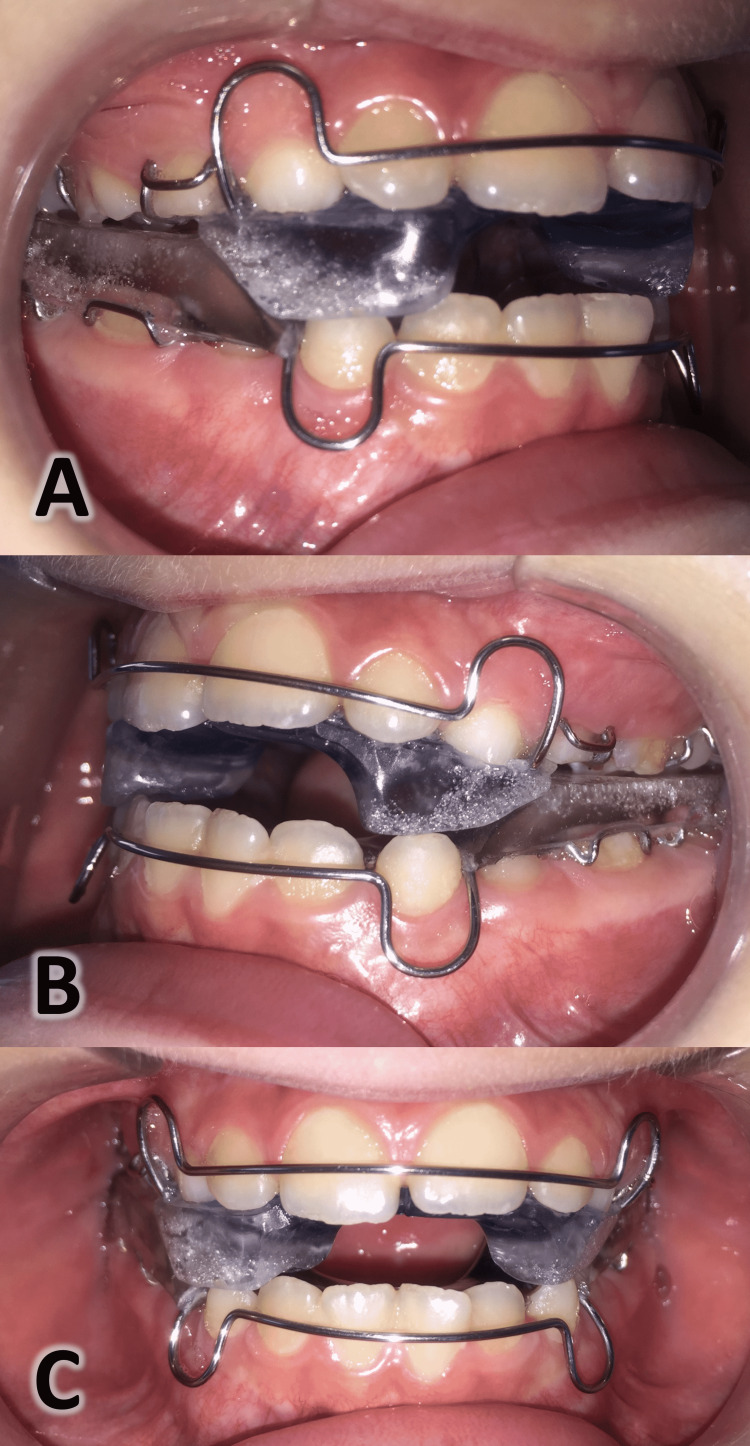
Intra-oral photographs of the employed appliance (the Reverse Twin Block appliance). A: right side view, B: left side view, C: frontal view.

All participating patients in this group were treated with an RTB appliance, as suggested by WJ Clark [28], fabricated at the maximum possible retrusion of the mandible with an inter-incisal clearance of 2 mm and a posterior vertical clearance of 5 mm. Bite blocks were inclined at 70 degrees to the occlusal plane in reverse configuration achieved by placing the upper block covering the deciduous molars anteriorly and enabling the lower block covering the lower molars to occlude behind it (Figure 2). The patients were instructed to wear the RTB appliance 22 hours a day except during meals. All patients were assessed every three weeks.

Reversed Twin Block + Photobiomodulation Therapy (RTB+PBMT) Group

In the RTB+PBMT group, the same steps followed in the control group were repeated regarding the use of the RTB. PBMT was applied as an adjunctive therapy using semiconductor gallium-aluminum-arsenide (Ga-Al-As) diode laser (Konftec; model Klas-dx 82, Taiwan), 808 nm wavelength in continuous mode, 250 milli-Watt power output, 5 Joules/cm^2^ energy density, 20 s per point application, employing a 5 mm diameter fiber optic tip, and total energy of 25 J per side, every visit. The laser parameters are shown in Table 1.

**Table 1:** Photobiomodulation therapy parameters used in the current trial mW: milliwatt, nm: nanometer, J\Cm^2^: Joule\Centimeters^2^, J: Joule, S: second, mm: millimeters

Laser type	GaAlAs Semiconductor	Time per point (S)	20 S
Wavelength (nm)	808nm continuous	Laser length (mm)	188 mm
Power (mW)	150-250 mW	Laser diameter (mm)	26 mm
Energy density(J/cm^2^)	5 J/cm^2^	Laser weight (g)	230 g
Total energy per side(J)	25J		
Frequency	on days 1, 3, 7, and 14 of the first month; afterward, every 15 days until the end of the treatment		

**Figure 3:**
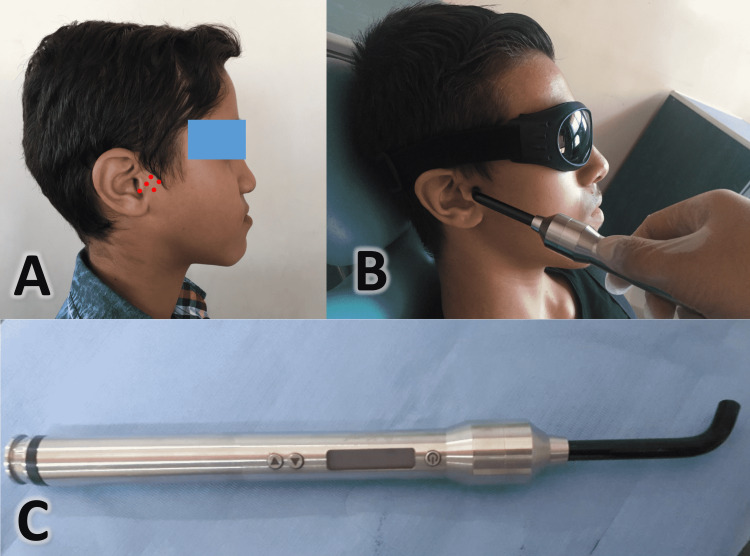
A patient in the experimental group where laser irradiation was applied. A: The application points in the TMJ region. B: Application of the photobiomodulation therapy (PBMT) at five points in the temporomandibular joint (TMJ) region. C: The Gallium–Aluminum–Arsenide semiconductor diode laser device used in the current study

Laser therapy was performed bilaterally in contact with the skin at five points (lateral, superior, anterior, posterior, and posterior-inferior points) located around the TMJ condyle, as shown in (Figure 3), on days 1, 3, 7, and 14 of the first month; and then every 15 days until the end of the treatment. All safety precautions were taken during laser application. The first author performed all clinical treatment procedures and laser applications. Cone-beam computed tomography (CBCT) images were taken before and after the end of treatment.

Outcome measures

The outcome measures of the current study were the 3D changes in the TMJ region and the skeletal changes following Class III treatment. CBCT images were obtained at the beginning of the treatment (T1) and after getting an adequate overjet of 2mm (T2). The CBCT images (Pax-i3D Green, Vatech, Seoul, Korea) were taken with a full field of view (i.e., a scan with 15 × 15 cm); voxel size of 0.2 × 0.2 mm; the radiologic parameters used were: 100 kVp, 10 mA, and 9 seconds scanning time. All patients were scanned with standard protocol: standardized head position, teeth in maximum intercuspation, and a horizontal plane parallel to the floor. The data obtained were exported to Digital Imaging and Communication in Medicine (DICOM) format.

Assessment of TMJ Changes

**Figure 4:**
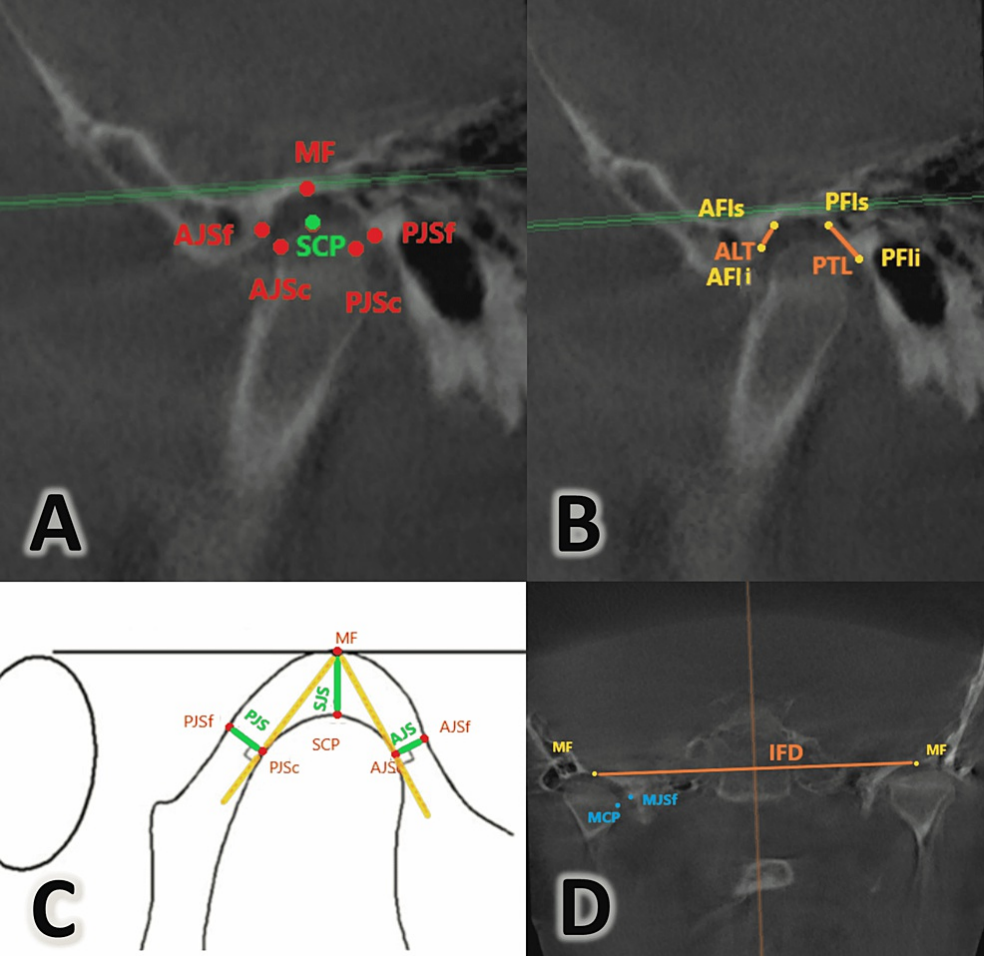
Measurements made on the Temporomandibular Joint A: MF: Bony mandibular fossa, PJSf: Posterior joint space “fossa point”, PJSc: Posterior joint space “condylar point”, AJSc: Anterior joint space “condylar point”, AJSf: Anterior joint space “fossa point”, SCP: Superior condylar point. B: AFIs: Anterior fossa “superior point”, AFIi: Anterior fossa “inferior point”, PFIs: Posterior fossa “superior point”, PFli: Posterior fossa “inferior point”, ALT: Anterior tuberculum inclination line, PTL: Posterior tuberculum inclination line. C: MF: Bony mandibular fossa, PJSf: Posterior joint space “fossa point”, PJSc: Posterior joint space “condylar point”, AJSc: Anterior joint space “condylar point”, AJSf: Anterior joint space “fossa point”, SCP: Superior condylar point, AJS: Anterior Joint Space, PJS: Posterior Joint Space, SJS: Superior Joint Space. D: MF: Bony mandibular fossa, MCP: Medial condylar point, MJSf: Medial joint space “fossa point”, IFD: Interfossae distance.

A set of linear measurements was evaluated within the temporomandibular joint region in the sagittal plane. Condyle volume was assessed using the measurement method adopted by Alhamadi and his colleagues [29], as shown in Table 2 and Figure 4.

**Table 2:** Definitions of the landmarks employed in the current study as well as the linear and angular measurements.

Sella (S)	The central point of the pituitary fossa in the middle cranial fossa
Nasion (N)	the most anterior point of the frontonasal suture in the midsagittal plane
Subspinale (point A)	The deepest midpoint on the anterior surface of maxilla
Supramentale (point B)	The deepest midpoint on the anterior surface of mandible
Maxillary sagittal position angle (SNA)	The angle between 3 landmarks: S, N, and A points, determining the anteroposterior position of the maxilla relative to the cranial base.
Mandibular sagittal position angle (SNB)	The angle between 3 landmarks S, N and B points, determining the anteroposterior position of the mandible relative to the cranial base.
Maxillo-mandibular sagittal relationship angle (ANB)	anteroposterior relation between maxilla and mandible relative to the anterior cranial base
Bony mandibular fossa (MF)	Centered mediolaterally bony of tuberculum and superiorly positioned
Superior condylar point (SCP)	The most right or left superior point of the condylar head.
Medial condylar point (MCP)	The most right or left medial point of the condylar head
Anterior joint space “fossa point” (AJSf)	The most posterior point of the right or left anterior wall of the mandibular fossa opposed to the shortest anterior condylar-fossa distance.
Anterior joint space “condylar point” (AJSc)	The most anterior point of the right or left condyle opposed to the shortest anterior condylar-fossa distance.
Posterior joint space “fossa point” (PJSf)	The most anterior point of the right or left posterior wall of the mandibular fossa opposed to the shortest posterior condylar-fossa distance.
Posterior joint space “condylar point” (PJSc)	The most posterior point of the right or left condyle opposed to the shortest posterior condylar-fossa distance
Soft tissue mandibular fossa (MFS)	The most superior and midpoint of the soft tissue right or left mandibular fossa region
Medial joint space “fossa point”(MJSf)	The most right or left lateral point of the medial wall of mandibular fossa
Anterior fossa “superior point” (AFIs)	Most prominent superior point of anterior inner wall of mandibular tuberculum in to construct anterior slop wall of mandibular tuberculum
Anterior fossa “inferior point” (AFli)	Most prominent inferior point of anterior inner wall mandibular tuberculum in to construct anterior slop wall of mandibular tuberculum
Posterior fossa “superior point”(PFls)	Most prominent superior point of the posterior inner wall of mandibular tuberculum to construct posterior slop wall of mandibular tuberculum.
Posterior fossa “inferior point”(PFli)	Most prominent inferior point of posterior inner wall mandibular tuberculum to construct posterior slop wall of mandibular tuberculum
Frankfort horizontal plane (HP)	Plane defined by three landmarks: right Porion, left Porion and left orbitale
Midsagittal plane (MSP)	Plane passing through Sella and Nasion points and perpendicular to the Frankfort horizontal plane
Anterior tuberculum inclination line (ALT)	The line between AFIS and AFIi points represents the anterior fossa inclination
Posterior tuberculum inclination line (PTL)	The line between PFIs and PFIi points represents the posterior fossa inclination line
Interfossae distance (IFD)	The distance between bony mandibular fossae (MF).
Anterior wall inclination to HP (ALT\HP)	The inner angle between ATL line and the Frankfort horizontal plane
Posterior wall inclination to HP (PTL\HP)	The inner angle between PTL line and the Frankfort horizontal plane
Anterior Joint Space (AJS)	The shortest distance between AJSc and AJSf.
Posterior Joint Space (PJS)	The shortest distance between PJSc and PJSf.
Superior Joint Space (SJS)	The shortest distance between SCP and MFS.
Medial Joint Space (MJS)	The shortest distance between MJSf and MCP.
Condyle volume (CV)	The volume of the condyle

**Figure 5:**
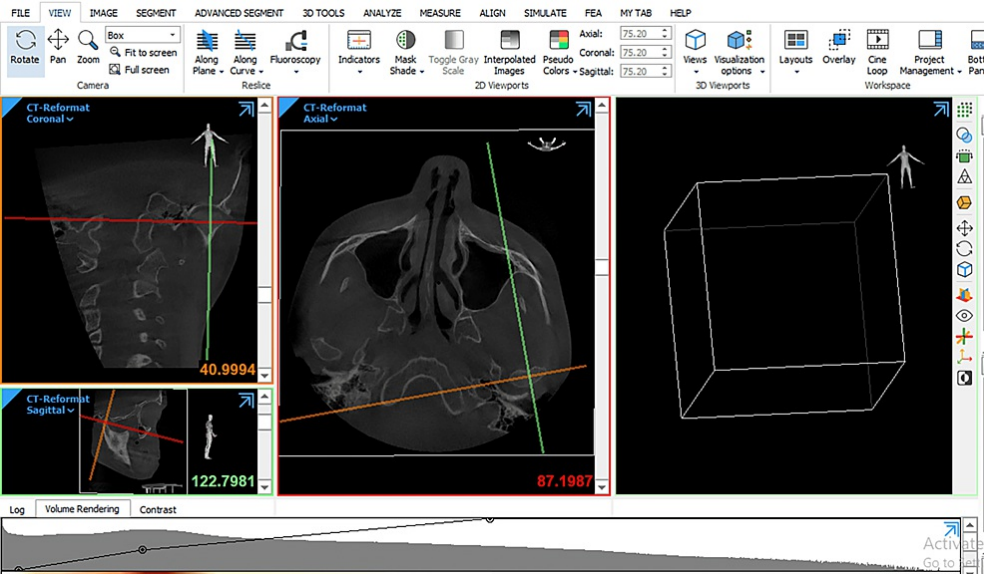
Cone-beam computed tomography (CBCT) images reorientation before reconstructing a 3D reproducible condyle model.

**Figure 6:**
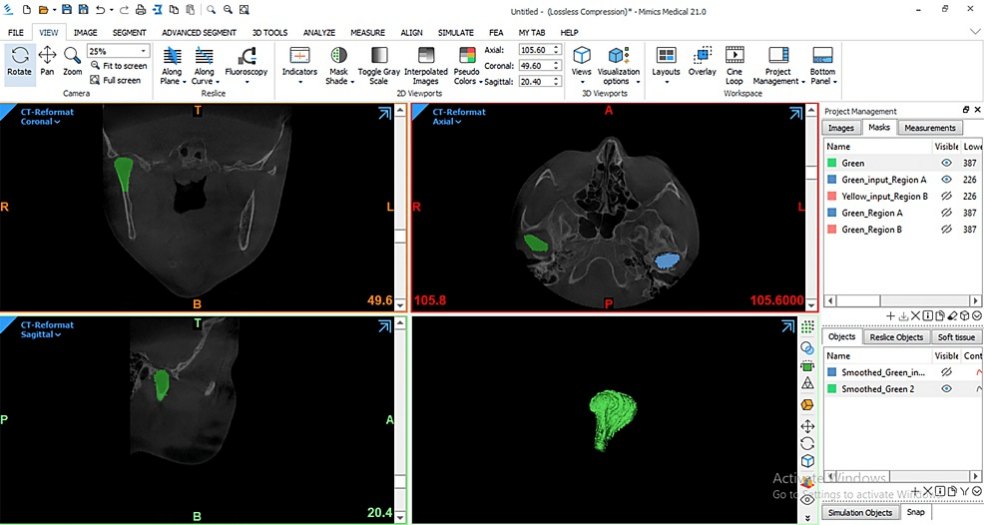
3D model of the condyle after being reconstructed in preparation for calculating its volume.

DICOM files were opened using Mimics 21.0 (Materialise NV Technologielaan, Leuven, Belgium). Then, the condylar head was segmented following the methods of a previous study [30], where the superior contour of the condyle on the coronal view was defined as the first radiopaque segment showing the beginning of the bone density of the condyle and the lateral borders to correspond to the segment that offers the largest possible amount of the condyle from all directions, while the lower boundaries were corresponding to where its section passed from an "ellipsoidal" shape to a more "circular" shape (Figure 5). Before condyle reconstruction, all other structures surrounding the condyle had to be isolated in all three planes of space. Each condyle was visualized in the recommended range of bone density and segmented using threshold tools. The remaining surrounding structures were removed using various sculpting tools. Then, 3-D multiplanar reconstructions were produced (Figure 6). Volumetric measurements were made for each condyle through the Mimics™ automatic function.

Assessment of the Skeletal Changes

Cephalometric images derived from CBCT images were used to assess sagittal skeletal changes in both groups. Landmarks and angular cephalometric measurements were defined according to Jacobson [31] and Riolo et al. [32].

The error of the method

Fifteen CBCT Images were randomly chosen from all images and re-measured one month after the first assessment. Reliability was evaluated using Intraclass correlation coefficients (ICC), which showed strong reliability ranging from 0.958 to 0.998. The systematic error was assessed with a paired t-test which showed no statistically significant differences between both measurements [33].

Statistical analysis

Statistical analysis was performed using the SPSS program (version 22.00; IBM Corp., Armonk, NY). The Kolmogorov-Smirnov test was used to test the normality of data distribution. The Chi-square test was used to detect intergroup differences in sex distribution, whereas the paired sample t-test was used to evaluate intergroup differences in age. Data were checked for pre-treatment equivalence using an independent t-test. The mean average for left and right TMJ sides was used. A paired t-test was used for intergroup comparisons of the measurements, whereas the independent t-test was used to compare the two groups. The significance level was set at 0.05.

## Results

Forty patients (26 males, 12 females; mean age 10.2± 0.81 years) were recruited and allocated randomly to either the RTB+PBMT group or the RTB-only group (Table 3). No dropout occurred, and complete follow-up and analysis were achieved for all patients. The mean treatment time was 258.68 ± 16.43 days in the RTB group and 193.53 ± 15.34 days in the RTB+PBMT group.

**Table 3:** Basic sample characteristics with regard to age and sex RTB + PBMT: Reversed twin block with photobiomodulation therapy; RTB: Reversed twin block. SD: standard deviation, ^a^ using Student t-test, ^b^: using Chi^2^ test

Group	RTB	RTB+PBMT	P-Value
Age years (mean± SD)	10.12±0.84	10.27±0.80	0.567^a^
Sex distribution: male/female	14/6	12/8	0.507^b^

TMJ changes

Inter-fossae distance increased significantly in each group (p<0.001; Table 4), with a statistically significant greater increase in the RTB group compared to the RTB+PBMT group (p<0.05; Table 5). No statistically significant differences were found in the inclination of the anterior and posterior glenoid fossae walls. Condylar volume increased significantly in the RTB group only (p<0.001). Both anterior and mediolateral joint spaces showed a statistically significant increase in both treatment groups (p<0.001), followed by a significant decrease in superior and posterior joint spaces in both groups (p<0.001). The greatest changes in joint spaces were observed in the RTB group (p<0.01).

**Table 4:** Descriptive statistics of the angular and linear measurements in each group as well as the P-values of significance testing for the observed change in each group. * Significant difference, RTB+PBMT: Reversed twin block with photobiomodulation therapy; RTB: Reversed twin block only; SD: standard deviation; SNA: Maxillary sagittal position angle; SNB: Mandibular sagittal position angle; ANB: Maxillo-mandibular sagittal relationship angle; HP: Frankfort horizontal plane; ALT\HP : Anterior wall inclination to HP; PTL\HP: Posterior wall inclination to HP; CV: Condyle volume; AJS: Anterior Joint Space; SJS: Superior Joint Space; PJS: Posterior Joint Space; MJS: Medial Joint Space

Variable	RTB (N=20)				RTB+PBMT (N=20)					
	T1		T2		P-Value	T1		T2		P-Value
	Mean	SD	Mean	SD		Mean	SD	Mean	SD	
SNA (°)	76.27	3.08	77.81	3.64	<0.001^*^	77.45	2.30	79.38	2.50	<0.001^*^
SNB (°)	77.46	3.08	76.58	3.81	0.015^*^	78.76	2.38	77.75	2.35	<0.001^*^
ANB (°)	-1.19	0.47	1.24	0.46	<0.001^*^	-1.30	0.59	1.62	0.52	<0.001^*^
FDI (mm)	91.06	3.32	91.78	3.14	<0.001^*^	89.27	2.33	89.71	2.34	<0.001^*^
ALT/HP (°)	43.65	7.66	43.98	7.44	0.377	47.65	8.22	47.17	08.52	0.060
PTL/HP(°)	59.37	8.21	59.62	8.23	0.200	59.2	7.79	58.94	7.74	0.117
CV (mm^3^)	2204.94	210.08	2492.92	254.77	<0.001^*^	2197.57	166.29	2260.50	160.58	0.224
AJS (mm)	1.79	0.27	2.05	0.32	<0.001^*^	1.91	0.46	2.02	0.44	<0.001^*^
SJS (mm)	2.92	0.46	2.65	0.49	<0.001^*^	2.89	0.34	2.72	0.37	<0.001^*^
PJS (mm)	3.01	0.47	2.59	0.47	<0.001^*^	2.67	0.63	2.55	0.60	<0.001^*^
MJS (mm)	2.26	0.30	2.59	0.30	<0.001^*^	2.26	0.25	2.44	0.23	<0.001^*^

**Table 5:** Descriptive statistics of the angular and linear changes that occurred in both groups as well as the p-values of significance testing between the two groups. * Significant difference; RTB+PBMT: Reversed twin block with photobiomodulation therapy; RTB: Reversed twin block only; SD: standard deviation; SNA: Maxillary sagittal position angle; SNB: Mandibular sagittal position angle; ANB: Maxillo-mandibular sagittal relationship angle; HP: Frankfort horizontal plane; ALT\HP : Anterior wall inclination to HP; PTL\HP: Posterior wall inclination to HP; CV: Condyle volume; AJS: Anterior Joint Space; SJS: Superior Joint Space; PJS: Posterior Joint Space; MJS: Medial Joint Space

Variable	RTB (N=20)		RTB+PBMT (N=20)		P-Value
	Mean change (T2-T1)	SD	Mean change (T2-T1)	SD	
SNA (°)	-1.54	1.42	-1.93	0.87	0.317
SNB (°)	0.88	1.47	1.002	0.90	0.762
ANB (°)	-2.43	0.63	-2.933	0.64	0.021^*^
FDI (mm)	-0.72	0.48	-0.43	0.22	0.033^*^
ALT\HP (°)	-0.34	1.68	0.47	1.05	0.082
PTL\HP (°)	-0.25	0.85	0.08	0.21	0.103
CV (mm^3^)	-287.97	264.92	-62.93	223.93	0.011^*^
AJS (mm)	-0.26	0.08	-0.11	0.08	0.001^*^
SJS (mm)	0.26	0.11	0.15	0.06	0.002^*^
PJS (mm)	0.42	0.07	0.11	0.08	<0.001^*^
MJS (mm)	-0.33	0.11	-0.18	0.08	0.001^*^

Skeletal changes

A statistically significant increase was found in the SNA angle in the two groups (p<0.001). The SNB angle decreased significantly in both groups (p<0.05 and P<0.001 for the RTB and RTB+PBMT groups, respectively). While a statistically significant increase was found in the ANB angle (p=0.001) in both groups, the most remarkable changes were found in the RTB+PBMT group (p<0.05).

Harms

No harm or untoward effects were observed during the course of this trial.

## Discussion

The RTB has been widely used in the early correction of Class III deformities [9,34]. Kidner et al. used the RTB effectively in growing Class III patients [9]. Similarly, studies by both Seehra et al. and Fareen et al. demonstrated the power of the RTB in the treatment of patients with Class III malocclusion [34,35]. However, the current literature lacks studies evaluating the effect of RTB treatment on the TMJs [10]. As expected, any changes in the occlusal features may impact both the morphology and the relationship between the TMJ structures [36]. Therefore, the present study focused basically on detecting the TMJ changes following the treatment.

Previous studies have used laser irradiation with different parameters of wavelength, energy, exposure time, and treatment sessions [37,38]. To the best of our knowledge, no previous study was conducted on humans using PBMT during Class III functional treatment. The present study used an Al-Ga-As diode laser with a wavelength of 808 nm, which had good penetration into the tissues and lay in the optimal wavelength range that provided a good photobiomodulation effect [39]. Irradiation dose is considered an important parameter when using PBMT [40]. Since no exact value has been defined, 5 joules/cm^2^ energy density with an output power of 250 mW on points was applied in this study as suggested by Brugnera et al. [41].

CBCT was used to assess 3D volumetric changes in the TMJ area during the growth. It was also considered an additional diagnostic tool when traditional radiographic methods failed to promote the accuracy of the studied structures [42]. According to TMJ 3D analysis, both groups were associated with a significant increase in the inter-fossae distance (RTB X=0.7 mm, RTB+PBMT X=0.43mm), with a significant difference between them. That could be attributed to both the treatment effect and spontaneous growth. These findings were similar to Lee's study [13], which reported that the treatment with a face mask caused an increment in the intercondylar distance. After treatment, neither group had a significant difference in anterior and posterior fossae wall inclination. In contra with these results, previous studies reported changes in anterior and posterior wall inclination caused by bone resorption of the posterior wall and bone apposition of the anterior one during treatment with a face mask and bone-anchored mini plate (BAMP), which may be a result of higher applied forces [13-15].

The current study showed increased condylar volume in both groups, but only the control group had significant changes. This difference may be due to both laser effects and growth. Since the treatment lasted longer in the control group than in the laser group, the changes resulting from the growth process may be more evident in this group. Laser with the current parameters may affect these findings. Although previous studies have proven the stimulation effect of LLLT on the condyle, Oksayan's study found deference in tissue response to different parameters [43]. Untreated subjects must be included to understand the natural growth effect on TMJ structures, which was not attainable in this study due to ethical reasons. To the best of our knowledge, no studies have evaluated condylar volumetric changes after treatment with RTB in Class III patients. Most studies compared condyle volume between different malocclusion types. Only Huqh et al. assessed the 3D effects of active skeletonized sutural distractor (ASSD) on the TMJ morphology of class III patients. They reported an increase in condyle volume with no statistically significant difference before and after treatment [44]. A comparison with this study may not be possible since ASSD conducts heavier forces (500g per side) than RTB, which may have inhibitory effects on condyle.

Regarding joint space changes, anterior and median joint spaces were significantly increased, followed by a significant decrease in both groups' posterior and superior joint spaces, indicating backward and upward condyle position after treatment. The most significant changes in joint spaces were observed in the control group. RTB treatment, laser usage, and treatment time, which lasted more in the control group, may cause a significant difference between the two groups. No previous study in the literature evaluated the 3D changes of TMJ after RTB treatment with and without PBMT. Few studies assessed the effect of other appliances on TMJ while treating class III patients. The present findings are consistent with El Feky and Rashid's conclusions [15], which reported a significant increase in anterior joint space and a decrease in both superior and posterior one after facemask treatment.

Similarly, Huqh's study observed increased anterior joint space, with a decrement in the posterior and superior one after treatment with ASSD appliance [44]. On the contrary, the CT study of Gong et al. found no significant increment in anterior joint space after the face mask treatment. But in parallel with current findings, they found a considerable decrement in superior and posterior joint spaces [45]. Yao et al.'s 2D study stated a significant increment of the anterior joint space followed by a decrement of the posterior joint space, which was correspondent with present results, but no significant changes in superior joint space, which was not compatible with the current study outcomes. This different conclusion might be due to the poor repeatability of cephalometric analysis [45].

In terms of skeletal changes, SNA increased significantly within the two groups after treatment with no significant difference between them, which Indicates a forward movement of the maxilla related to the cranial base. This was in line with Seehra et al.'s [34] and Minase et al.'s [46] results, who reported an improvement in SNA after treatment with RTB and Modified RTB, respectively. But this was contrasting with Kidner et al. study, which stated no important changes in SNA After treatment with RTB [9]. There was a statistically significant decrease in the mean value of SNB in the RTB group (x=0.88°) and RTB+PBMT (x=1°), without any significant difference between the two groups. This decrement could be explained due to the backward and downward rotation of the lower jaw. This was in agreement with the previous studies of Minase et al. and Kidner et al., who found a decrease in SNB angle after treatment with RTB and modified RTB, respectively [46,9]. The ANB angle improved significantly within the two groups (RTB group x= 2.43°, RTB + PBMT group x= 2.93°), and that may be attributed to the sagittal maxilla improvement with the cranial base and the downward rotation of the lower jaw, but the most significant changes were found in RTB+PBMT group, This difference may be caused by the short duration of treatment in the RTB+PBMT group. These findings were in contrast to the Kidner et al. study [9], which mentioned minor changes in ANB. This difference could be attributed to the small sample size in their study. In contrast, similar positive results were indicated by Majanni and Hajeer after removable mandibular retractor treatment [47].

Limitations

Both the short evaluation period and the absence of an untreated group were the main limitations of the current study. Moreover, this study did not evaluate the role of gender in TMJ changes. Therefore, further randomized controlled studies with long-term observation and different PBMT parameters should be carried out.

## Conclusions

No significant effects on the condylar position were found following skeletal class III treatment with a reversed twin block appliance in conjunction with the photobiomodulation therapy. A small difference in condylar volume was noticed between the RTB group and the RTB+PBMT group. Additional randomized controlled trials are needed with various irradiation parameters and protocols to arrive at better conclusions.
